# Vitamins, Vegetables and Metal Elements Are Positively Associated with Breast Milk Oligosaccharide Composition among Mothers in Tianjin, China

**DOI:** 10.3390/nu14194131

**Published:** 2022-10-04

**Authors:** Xinyang Li, Yingyi Mao, Shuang Liu, Jin Wang, Xiang Li, Yanrong Zhao, David R. Hill, Shuo Wang

**Affiliations:** 1 Tianjin Key Laboratory of Food Science and Health, School of Medicine, Nankai University, Tianjin 300071, China; 2 Abbott Nutrition Research & Development Center, Abbott Ltd., Shanghai 200233, China; 3 Abbott Nutrition, Columbus, OH 43219, USA

**Keywords:** human milk oligosaccharides, diet, vegetable, vitamin, nutrients

## Abstract

Background: Human milk oligosaccharides (HMOs) are a group of breast milk carbohydrates exerting pivotal benefits for breastfed infants. Whether maternal diet is associated with breastmilk HMO composition has not been well-characterized. Objectives: We investigated the associations between dietary nutrient intake and HMO concentrations in a general pregnant and postpartum population. Methods: A total of 383 breast milk samples and the corresponding food frequency questionnaires during 0–400 postpartum days from 277 mothers were collected. Six different HMOs were detected in mothers’ milk. The correlation between nutrients and HMOs were analyzed using a linear mixed-effects model. Results: We found plant nutrients, vitamin A, vitamin C and vegetables as positive predictors of 3-fucosyllactose; vitamin B1 and vitamin B2 were positive predictors for 2′-fucosyllactose level and the sum of 2′-fucosyllactose and 3-fucosyllactose; tocopherol and metal elements were positive predictors for 3′-sialyllactose; and metal elements were positively associated with the sum of all the six HMOs; the milk and lactose intake was a positive predictor of lacto-N-tetraose levels and the sum of lacto-N-tetraose and lacto-N-neotetraose. Conclusions: The results show that vegetables, vitamins and metal elements are dietary components positively associated with HMO concentrations.

## 1. Introduction

Breast milk contains a wide variety of nutrients to comprehensively support age-appropriate growth in early life [1]. The World Health Organization recommends exclusive breastfeeding in the first six months and continued breastfeeding until the age of two or beyond [2]. Human milk oligosaccharides (HMOs) are the third most abundant component in breast milk. As non-digestible carbohydrates, HMOs play roles in supporting the developing immune system of infants [3]. HMOs are selective substrates for intestinal microbiota, such as Bifidobacterium and Bacteroides, and inhibit the colonization of pathogens by acting as decoys for surface carbohydrates on intestinal epithelial cells and therefore inhibit the binding of pathogens and support immune maturation by directly interacting with immune cells. However, human milk has a considerably higher concentration of oligosaccharides than milk from other animals [4].

Diet is an important factor that influences the composition of human milk and therefore contributes to the health of both mother and infant. Several nutrients in human milk are closely correlated with the intake of the nutrient [5]. Whether HMO concentrations are influenced by maternal diet is unclear. Studies in cows have shown that diet might be a factor in determining bovine milk oligosaccharide concentration [6]. However, milk oligosaccharides are much higher in human milk than bovine milk and may vary between individuals. In one observational study, concentration of all HMOs varied 3.7-fold, and the 19 individual HMOs measured each varied from 20 to >100-fold from person to person at 3–4 months postpartum. Multiple characteristics beyond genetic secretor status were associated with the concentration of some HMOs, but diet quality was not identified as a significant factor affecting HMO concentration [7]. Another study of Brazilian mothers at 17 to 76 days postpartum also found that the concentration of HMOs varied from person to person, and diet was not a significant predictor of HMO levels [8]. Moreover, our previous study in a cohort of mothers from 0–400 day postpartum also showed that HMO concentrations vary greatly according to stage of lactation and secretor genotype [9]. Therefore, additional research is needed in order to clarify the effect of nutrition on maternal HMO composition, especially in Chinese populations.

In the current study, HMO concentrations and dietary patterns were examined among healthy pregnant women in Tianjin, China. Our objective was to explore the association between diet and HMO concentration using a prospective cohort study conducted in a general pregnant population. This observational study provides new evidence that several dietary nutrients may be potential factors shaping HMO concentrations among lactating mothers.

## 2. Materials and Methods

### 2.1. Study Population

The study population was recruited on 10 November 2017 to 07 December 2018, from antenatal clinics and classes at Tianjin Hospital of ITCWM Nankai Hospital, Tianjin, China, which is a part of an observational cohort study approved by the China Clinical Trial Center (ChiCTR1800015387). A total number of 277 healthy women were consent to participate in the study. Breast milk samples were collected at 5 different stages: Colostral milk 0–5 days post-partum, transitory milk 10–15 days post-partum, mature milk 40–45 days post-partum, mature milk 200–240 days post-partum and mature milk 300–400 days post-partum. The demographic information and dietary questionnaire data of mothers and infants were also collected at the same time when milk was sampled. Data were collected by uniformly trained hospital doctors and medical students. The inclusion criteria were that the pregnant women had lived in the area for more than two years, were aged 20–35 years, planned to breastfeed for more than 3 months, had singleton pregnancies and had a gestational age of 37–42 weeks. Subjects were excluded if the mother–infant pair had any health conditions, including chronic diseases, low Apgar score (<8) for neonates, presence of acute or chronic infectious disease, or use of prescription drugs during pregnancy or lactation that could affect nutrient metabolism. This study was conducted according to the guidelines laid out in the Declaration of Helsinki, and all procedures involving human subjects were approved by the Ethics Committee of Clifford Hospital and registered in the China Clinical Trial Center (ChiCTR1800015387). Written informed consent was obtained from all participants.

### 2.2. Milk Sample Collection

A standardized sampling procedure was used for all mothers. All mothers were asked to empty the milk from one breast between 8:00–11:00 am on the day of collection using a manual or electric breast pump. Breastmilk was mixed thoroughly and collection tubes were inverted 6 times and 30–50 mL was poured into sterile light-proof centrifuge tubes. Except on days 0–5 and 10–15 postpartum, when we collected 8–10 and 10–30 mL, respectively. The remaining breast milk was returned to the mother for feeding the baby. All breast milk samples were transported to Abbott Nutrition Research and Development Centre, Shanghai, China and stored at a constant temperature (−80 °C) during transport and storage for later analysis.

### 2.3. HMO Detection

HMOs in breast milk was detected by high-performance anion exchange chromatography with pulsed amperometric detector (HPAEC-PAD) as described in previous studies [6,9].

### 2.4. Nutrients Contents from Food Frequency Questionnaire (FFQ)

Dietary data were collected by performing a food frequency questionnaire (FFQ) survey with a validated questionnaire [10] among women on the day of milk collection. Nutrient data were calculated by using the China Food Composition Tables (CFCT, 2009 edition) [11]. For food items not included in the CFCT, the International Food Composition Resources from the USA Food and Nutrition Information Center was employed. Finally, all 82 nutrient data items, including total energy, the proportion of protein, fat and carbohydrate macronutrient, and other 78 individual nutritional items, were calculated. The list of all 82 nutrient data items evaluated are shown in Supplementary Table S1.

### 2.5. Energy Adjustment for Diet

Two methods, the nutrient density method and the residual method, were employed for energy adjustment of micronutrients data. For macronutrients (carbohydrate, protein and fat), the percentage (%) of total energy was presented. For the remaining 79 micronutrients items, the values were adjusted with energy using the following formula:

$$\mathrm{density}\;=\frac{\mathrm{micronutrients}}{\mathrm{energy}}\times \mathrm{mean}\;\mathrm{of}\;\mathrm{micronutrients}$$

residual=v+∣minv∣

v is the residual of the linear regression of the micronutrients for energy.

Principal component analysis was further performed and the result of a scree plot with parallel analysis was used for sensitivity analysis.

### 2.6. Principal Component Analysis

To better construct a model for the relationship between HMO and nutrients, principal component analysis (PCA) was performed for adjusted dietary data. A scree plot with parallel analysis was performed to determine the optimal energy adjustment method, and the number of principal components was determined using the criteria of cumulative explained variance ratio >80%. Then, the factor loading matrix was calculated and transformed, and the communality of each principal component for nutrients was calculated, and the loading factors of principal components for each entry were transformed and merged with each entry and used for further linear mixed-effects model analysis.

### 2.7. Mixed-Effect Model

We used a linear mixed-effects model to explore the critical principal components of diet for predicting HMO concentrations, and included adjustment for the random effects of non-dietary factors. The mixed-effect model assumed a beta response distribution for the relative abundance response, with participant as a random effect and compound symmetric variance–covariance error structure. The dependent variables included the 13 principal components, and independent variables were the stages of lactation and Secretor status. Here, we performed a linear mixed-effects model analysis for each of the 6 HMOs, and the sum (the sum of the 6 HMOs), FL (the sum of 2′-FL and 3-FL), LT (the sum of LNT and LNnT) and SL (the sum of 3′-SL and 6′-SL) variables were also evaluated for the effect of diet in the model by using the linear mixed-effects model.

### 2.8. Statistical Analysis and Plotting

All exploratory and descriptive statistical analyses were performed with the use of R version 4.0.5. HMO concentrations were reported as the median (percentile 25, percentile 75) since some of the data presented a non-normal distribution. The variation of HMO concentrations over five lactational stages was explored using an independent non-parametric test (Kruskal–Wallis test, all pairwise). The milk samples were separated into two groups according to the distribution of the 2′-FL level: Low and High 2′-FL concentration groups. The HMO levels in the two groups were compared by Student’s *t*-test. A Pearson correlation test was performed between HMOs concentration and baseline characteristics in each subgroup. All statistical analyses were considered significant at *p* < 0.05 (two-sided). The sum of the concentrations of 2′-FL, 3-FL, LNT, LNnT, 3′-SL and 6′-SL represented the total HMO concentrations. Principal component analysis was carried out with the R package Psych (version 2.1.9). The mixed-effect model was carried out with R package lmerTest (version 3.1-3) [12]. Figures were generated by using R version 4.0.5 with R packages ggplot2 (version 3.3.5), ggstatsplot (version 0.9.0) [13] and ggpubr (version 0.4.0). The *p* < 0.05 was considered significant unless otherwise specified.

## 3. Results

### 3.1. Baseline Characteristics

The women enrolled in the study were healthy and had adequate dietary intake. Table 1 describes the baseline characteristics of the women included in this study. Dietary intake of all 82 nutrient items is shown in Supplementary Tables S1 and S2. After performing the Pearson correlation test in each subgroup, we found that the baseline characteristics were not correlated with HMO concentration.

### 3.2. HMO Levels Are Significantly Different between Secretors and Non-Secretors

Secretor status was defined by the presence of 2′-FL > 200 (mg/L), and all milk samples were categorized into secretors (2′-FL > 200 mg/L) and non-secretors (2′-FL ≤ 200 mg/L). We found that the level of each HMO was significantly different between secretors and non-secretors except for 6′-SL and 3′-SL (Table 2). The result suggests that secretor phenotype or non-secretor phenotype is a critical factor for determining HMO concentration.

### 3.3. HMOs Level Are Significantly Different between Different Stages

We then explored HMO concentrations according to different postpartum stages of lactation: 0–5 days (CM, Colostral Milk), 10–15 days (TM, Transitory Milk), 40–45 days (MM, Mature Milk), 200–240 days (M2, Mature milk 2) and 300–400 days (M3, Mature Milk 3) post-partum. We found that all HMOs varied significantly across each stage of lactation in both secretor milk (*n* = 289, Table 3) and non-secretor milk (*n* = 94, Table 4). As described in our previous study on mothers from South China [6], HMO concentrations vary across the stages of lactation according to known patterns. These results suggest that lactation stage is a significant factor in determining HMO concentration in breastmilk.

### 3.4. Model for HMOs and Components

By performing PCA, we found that the number of components was 13 for residual data, while the number of components was 14 for density data. Therefore, we used the residual model for energy adjustment. In this model, all the 13 principal components (PC1–PC13) had an eigenvalue >1 and were therefore included in the mixed-effect model as fixed effects. All six HMOs and summed HMO data, and secretor phenotype were designated as the random effectors. Our results show that several principal components, PC1, PC4, PC5, PC7, PC10, PC11, and PC12 are significant factors predicting HMO concentration. Table 5 shows the beta value and *p* value of PCs for each HMO, and Table 6 shows the top five nutrients for these PCs significantly correlated with HMOs.

## 4. Discussion

Here, we found that almost all the significant correlated dietary PCs with HMOs in the model were positive correlations, suggesting a positive effect of diet on HMO concentration. This is in alignment with the World Health Organization (WHO) guideline for pregnant woman that a healthy diet consists of a variety of foods [14].

Given that HMO only present in milk and cannot be obtained from natural sources, the in vivo biosynthesis of HMOs is critically important [15]. However, the mechanism of HMO biosynthesis in vivo is still largely unknown [16]. At present, it is known that HMOs are synthesized by extending lactose with other carbohydrates at the non-reducing ends in reactions catalyzed by glycosyltransferases in the mammary gland [3,17]. HMOs are biosynthesized by non-template-directed process glycosyltransferase [18]. Therefore, we hypothesize that in cases where adequate nutrition is present the activity of glycosyltransferases may be a key factor for enhancing HMO concentration. In our study, we found that multiple vitamins and metal ions are positively associated with HMO concentration. In fact, both vitamins and metals are important for the function of enzymes. Multiple vitamins serve as coenzymes, such as vitamin B1 and vitamin B2, and metal ions are also essential for enzymatic activity and may incorporate metal ions into the active site or rely on metals for enzyme activation [19]. Whether these nutrients affect HMO concentration by enhancing glycosyltransferase activity and characterization of their mechanism of action is of interest for future research.

In our study, we found that PC10 (mainly vitamin B supplement) is a positive dietary factor associated with 2′-FL and the sum of 2′-FL and 3-FL. PC11 (mainly vitamin A and vitamin C) and PC12 (vitamin B3) emerged as the positive predictive factor for 3-FL. A most recent study also pointed out that vitamin intake was associated with HMOs levels [20]. Our results, in accordance with the previous study, both suggest that vitamin supplements might be a positive nutrition factor to enhance milk HMO concentration in lactating mothers. Interestingly, by analyzing the sum of all six HMOs, we found that PC1 (metal) is the most significant predictive factor for the sum concentration of all HMOs in the mixed-effect model. PC1 was not a significant predictor of any single HMO in our study, suggesting that metal may promote a wide range of HMO concentrations, and may work through a different mechanism than other nutrients which seem to be associated with individual HMOs.

Apart from vitamins and metals, we found that vegetables (PC4 and PC11) are also positively associated with 3-FL. This may be due to the high concentrations of vitamins found in vegetables. Moreover, other benefits of eating more vegetables may also contribute to enhanced HMO content of breastmilk. In 2019, The Lancet Commission emphasized the broad benefits of eating vegetables [20]. A recent study also found that higher vegetable intake in mothers is associated with a lower incidence of allergy in offspring [21]. Our results suggest a beneficial role of eating vegetables with regards to HMO levels in breastmilk. In fact, China consumes the largest amount of vegetables per capita of any country [22]. Even so, we still identified a positive correlation between vegetable intake and 3-FL concentration in breastmilk, suggesting that there may be additional benefits for increasing vegetable consumption.

It is worth noting that cookie consumption, from PC12, was the only factor negatively correlated with HMO concentration in our study. The statistical significance between 3-FL and PC12 in this model is *p* < 0.1 and the loading factors of PC12 with cookie was −0.538, suggesting that high-sugar and high-fat foods were negatively associated with the HMO concentrations in breast milk. This is the first time we point out a negative correlation between high-sugar and high-fat foods with HMOs but the plausible mechanism and possible confounding factors need further research. Furthermore, we observed another marginal correlation between lactose intake (PC7) and LNT and the sum of LNT and LNnT. These results seems reasonable because the synthesis of LNT or LNnT requires one more unit of lactose as N-acetyllactosamine (Gal-β-1,4-GIcNAc) than the fucosylated HMOs or sialylated HMOs [7]. The synthesis of fucosylated HMOs and sialylated HMOs also requires one lactose (Gal-β-1,4-Glc) but was not associated with dietary lactose intake [23,24]. Regardless, the impact of dietary lactose on HMO profiles needs more research.

In the current study, we use a mixed-effect model to explore the correlation between nutrients and HMOs. In fact, HMOs vary largely across different stages of lactation and according to secretor status [25]. Diet may influence HMO concentration at time points that may not have been reflected in our questionnaire. Therefore, the mixed-effect model is well-suitable for evaluating the potential effects of dietary nutrients on HMO concentrations in breastmilk [26]. Of note, Nakagawa et al. (2017) proposed a method to compute marginal and conditional r-squared values for the mixed-effect model [27]. For mixed models, the marginal r-squared considers only the variance of the fixed effects, while the conditional r-squared takes both the fixed and random effects into account. Nakagawa et al.’s novel method made it possible for us to compare the relative importance of nutrients and random effects. Our study showed that although the 13 principal components (PC1–PC13) represent more than 80% of the variance in the diet, they merely accounted for a total of 0.3–1.6% of the HMO variance, with random effects, the lactation stage and secretor status, accounting for the majority of the variance in HMO levels (Table 5). This suggests that HMO synthesis and concentration are still largely dependent on gene regulation but not non-genetic factors. This is in accordance with a previous study [7].

The previous study by Azad et al. in 2018 [7] investigated a number of factors that may be associated with HMOs in the Canadian population. In Azad et al.’s study, factors were divided into environmental factors (season and city), nonmodifiable factors (secretor status, lactation stage, ethnicity, etc.) and modifiable factors (diet, delivery mode, and BMI, etc.). The authors found that secretor status and lactation stages were strongly associated with most individual HMO concentrations, which is in accordance with our current study and previous study [9] that HMOs concentrations varies largely between secretors and non-secretors, and different stages (Table 2 and Table 3). Additionally, our investigation with the mixed-effect model also suggests that gene regulation was still the major factor for HMOs variance (Table 5), which is in agreement with Azad et al.’s conclusion. However, there are differences between our study and Azad et al.’s. The present study focused more on the details of diet by calculating the food items into more than 80 specific nutrients (supplementary Table S1) with calculation of food composition. Furthermore, energy adjustment and the principal component analysis on the nutrients were then performed before the correlation analysis with HMOs, which provided detailed information on dietary nutrients rather than Azad et al.’s diet quality scores and food items. Admittedly, Azad et al.’s research demonstrates a far more complex spectrum of factors and populations and more kinds of HMOs than our study [7]. These are the limitations of our research. In the future, more factors other than diet may play potential roles in HMO concentration that need further research.

Although only six HMOs were included in our study, these six HMOs represent 40% of existing HMOs [28], and cover all six major types of HMOs: fucosylated, sialylated and acetylated HMOs [29,30]. In addition, we found that several nutrient items, such as the total energy, increased gradually from 1929.1 kcal at 0–15 days to 2200–2300 kcal after 40 days (Supplementary Table S2, the diet between different stages), which is different in comparison with other studies and may affect our conclusions [31,32]. This may be because local culture from Tianjin advises that “the nourishment after birth should be gradual and gentle (清调补养)”. It has been reported that energy intake may affect the HMO levels and multiple breastfeeding behaviors [33,34]. Although our results do not show a significant correlation between energy intake and HMOs, whether the particular dietary habit may influence HMOs concentration needs further research. In the future, nationwide multi-center surveys should be applied to rule out the potential bias of one location, and more HMOs should be included to better explore the potential relationship between nutrition and HMO content of breastmilk.

## 5. Conclusions

In conclusion, this is the first report demonstrating that dietary intake of vitamins and metal elements, as well as vegetables and lactose, are correlated with breastmilk HMO concentrations based on an observational study through all the lactation periods (0–400 days) among lactating women in Tianjin, China. These findings support the adoption of good dietary practices during pregnancy and lactation. Potential mechanistic connections between dietary nutrients and HMO synthesis need further research and this approach should be expanded to examine additional HMO structures in the future.

## Figures and Tables

**Table 1:** The baseline characteristics of participants.

Baseline Characteristics	Mean (q25, q75) or n Count
Mothers enrolled	277
Age (y)	30.15 (28, 32)
Number of pregnancies	1.52 (1, 2)
Number of deliveries	1.27 (1, 2)
c-section/natural	84/193
Pre-pregnancy weight (kg)	58.62 (52.5, 63)
Prepartum weight (kg)	73.1 (67, 79)
Weight gain (kg)	14.24 (11, 18)
Height (m)	1.64 (1.6, 1.67)
Pre-pregnancy BMI	21.91 (20.03, 23.44)
Prepartum BMI	27.29 (25.1, 29.3)
Infant sex, male/female	141/135
Education ^1^	3.04 (3, 3.5)

^1^ Education: 1 primary school, 2 middle school, 3 high school, 4 university or college; Data are expressed as mean (percentile 25, percentile 75) or counts.

**Table 2:** The HMOs between secretors and non-secretors.

HMO	Secretor Milk (*n* Sample = 289)	Non-Secretor Milk (*n* Sample = 94)	*p* Value (adj. *t*-Test)
3-FL (mg/L)	469.11 (228.54, 1231.22)	1384.59 (837.39, 2073.68)	8.96 × 10^−14^
2′-FL (mg/L)	1809.11 (1106.02, 2930.43)	24.67 (13.42, 48.15)	1.08 × 10^−80^
LNnT (mg/L)	114.31 (51.91, 231.05)	50.71 (18.04, 153.2)	0.002376952
LNT (mg/L)	435.58 (257.37, 691.05)	898.87 (383.78, 1610.93)	1.97 × 10^−8^
6′-SL (mg/L)	182.56 (28.43, 430.39)	206.70 (26.13, 418.84)	0.54555129
3′-SL (mg/L)	135.48 (102.76, 203.94)	126.71 (92.86, 188.45)	0.13571986
FL (mg/L)	2610.31 (2243.56, 3237.48)	1417.10 (872.09, 2104.38)	2.06 × 10^−29^
SL (mg/L)	298.79 (163.86, 649.54)	293.67 (143.16, 613.62)	0.353030878
LT (mg/L)	595.4 (323.03, 924.43)	961.21 (403.02, 1861.66)	2.22 × 10^−6^
sum (mg/L)	3513 (2902.41, 5053.34)	3005.08 (2637.14, 3692.77)	7.85 × 10^−11^

FL: the sum of 3-FL and 2′-FL; SL: the sum of 6′-SL and 3′-SL; LT: the sum of LNnT and LNT; sum: the sum of all the six kinds of HMOs; Data are expressed as median (percentile 25, percentile 75).

**Table 3:** The HMOs between different stages in secretors (*n* = 289).

HMO	CM (*n* = 75)	TM (*n* = 16)	MM (*n* = 68)	M2 (*n* = 72)	M3 (*n* = 58)	*p* Value (Kruskal–Wallis Test)
3-FL (mg/L)	176.26 (119.82, 294.18)	201.47 (137.66, 327.16)	377.18 (234.7, 487.84)	1213.61 (893.13, 1493.38)	1393.44 (1028.57, 1640.19)	4.33 × 10^−42^
2′-FL (mg/L)	3537.05 (3005.28, 4366.29)	2689.71 (2342.02, 3019.01)	2009.38 (1566.06, 2544.99)	1193.59 (862.31, 1526.61)	1006.85 (675.33, 1282.08)	1.02 × 10^−39^
LNnT (mg/L)	293.62 (246.06, 414.73)	216.35 (174.76, 301.17)	117.55 (74.26, 164.57)	58.92 (37.17, 94.25)	37.83 (19.55, 63.11)	1.72 × 10^−41^
LNT (mg/L)	656.47 (456.51, 1099.37)	1261.48 (943.38, 1512.82)	534.04 (348.77, 774.67)	254.56 (198.96, 352.79)	297.9 (182.05, 404.29)	2.27 × 10^−24^
6′-SL (mg/L)	464.68 (391.03, 548.36)	552.09 (493.79, 756.52)	294.43 (180.98, 402.07)	32.38 (20.82, 50.21)	20 (10.62, 33.46)	4.66 × 10^−48^
3′-SL (mg/L)	253.69 (202.9, 315.39)	153.48 (131.16, 199.67)	105.39 (86.19, 118.91)	114.95 (93.71, 139.61)	139.54 (103.72, 163.91)	5.24 × 10^−30^
FL	3697.9 (3186.22, 4621.85)	2921.89 (2605.74, 3278.7)	2504.21 (1988.17, 3007.23)	2372.99 (2180.82, 2609.96)	2373.63 (2120.77, 2582.83)	8.61 × 10^−25^
SL	717.46 (649.54, 812.08)	737.02 (645.97, 908.78)	414.95 (278.87, 511.85)	154.86 (135.26, 187.18)	164.51 (131.33, 202)	4.74 × 10^−47^
LT	1047.8 (768.11, 1382.07)	1537.79 (1186.76, 1760.79)	683.8 (500.42, 907.64)	291.53 (249.25, 434.18)	334 (219.81, 485.35)	8.46 × 10^−35^
sum	5755.93 (5187.86, 6402.73)	5142.36 (4793.08, 5678.31)	3690.99 (3119.37, 4075.24)	2930.6 (2752.11, 3165.77)	2934.16 (2638.05, 3253.69)	1.53 × 10^−41^

FL: the sum of 3-FL and 2′-FL; SL: the sum of 6′-SL and 3′-SL; LT: the sum of LNnT and LNT; sum: the sum of all the six kinds of HMOs; Data are expressed as median (percentile 25, percentile 75).

**Table 4:** The HMOs between different stages in non-secretors (*n* = 94).

HMO	CM (*n* = 25)	TM (*n* = 4)	MM (*n* = 23)	M2 (*n* = 21)	M3 (*n* = 21)	*p* Value (Kruskal–Wallis Test)
3-FL (mg/L)	696.47 (580.34, 961.76)	751.45 (629.03, 898.64)	1117.73 (857.49, 1448.78)	2179.61 (1638.99, 2474.44)	2050.38 (1692.3, 2210.65)	1.92 × 10^−11^
2′-FL (mg/L)	69.35 (54.45, 93.84)	14.87 (8.12, 25.83)	30.12 (25.2, 39.36)	15.25 (12.2, 22.08)	13.43 (10.75, 19.17)	1.20 × 10^−9^
LNnT (mg/L)	282.99 (206.1, 368.4)	74.24 (55.22, 89.74)	64.52 (40.17, 94.88)	19.02 (13.12, 30.15)	14.23 (7.13, 26.45)	9.83 × 10^−15^
LNT (mg/L)	2120.76 (1616.91, 2555.86)	2589.37 (2164.01, 3227.1)	1065.83 (821.18, 1408.14)	284.08 (175.62, 606.28)	375.25 (261.77, 567.98)	6.07 × 10^−13^
6′-SL (mg/L)	451.66 (400.91, 488.5)	745.42 (544.11, 900)	274.53 (184.17, 370.54)	31.77 (11.49, 45.03)	17.48 (9.14, 24.11)	4.21 × 10^−15^
3′-SL (mg/L)	239.43 (222.49, 300.21)	150.77 (131.12, 173.99)	97.62 (74.04, 122.7)	100.22 (83.6, 112.25)	123.2 (91.85, 134.96)	2.63 × 10^−11^
FL	777.62 (650.94, 972.71)	759.53 (644.69, 910.14)	1126.91 (899.39, 1481.16)	2191.46 (1653.96, 2490.18)	2068.14 (1733.74, 2222.3)	3.62 × 10^−11^
SL	709.06 (618.19, 768.08)	903.32 (671.43, 1084.91)	378.15 (279.82, 474.28)	142.74 (119.33, 162.85)	146.43 (114.23, 163.69)	4.88 × 10^−14^
LT	2508.49 (1923.8, 2791.45)	2638.76 (2231.66, 3279.55)	1140.5 (865.38, 1484.22)	304 (191.83, 640.3)	401.7 (279.79, 581.82)	1.17 × 10^−13^
sum	3927.75 (3646.62, 4172.64)	4535.04 (3853.88, 5201.93)	2727.3 (2560.26, 3120.88)	2809.38 (2533.22, 3097.37)	2687.3 (2478.14, 2946.63)	3.77 × 10^−9^

FL: the sum of 3-FL and 2′-FL; SL: the sum of 6′-SL and 3′-SL; LT: the sum of LNnT and LNT; sum: the sum of all the six kinds of HMOs; Data are expressed as median (percentile 25, percentile 75).

**Table 5:** The linear mixed-effects analysis of diet principal components for HMOs (beta value).

	3-FL	2′-FL	LNnT	LNT	6′-SL	3′-SL	FL	LT	SL	Sum
PC1	26.29	31.62	−2.44	18.72	1.67	5 #	59.82	16.37	6.63	80.733 *
PC2	−12.06	32.68	0.54	15.31	0.84	−0.63	19.54	16.13	0.64	37.179
PC3	1.53	34.12	−2.27	−8.31	4.05	−0.06	34.3	−10.31	4.31	29.749
PC4	54.06 **	−25.52	−3.1	9.56	−7.98	4.01	29.18	6.21	−4.14	30.238
PC5	−18.21	16.12	5	−15.65	2.39	4.93 #	−0.14	−10.91	7.02	−6.476
PC6	−7.63	−16.7	−4.62	0.02	8.46	−2.69	−27.23	−5	5.93	−24.371
PC7	−26.49	−10.19	0.31	44.26 #	1.17	1.27	−35.3	44.67 #	2.47	10.338
PC8	17.44	6.08	−4.31	30.03	3.07	−0.12	26.08	25.63	2.85	51.341
PC9	10.27	−39.06	−1.65	19.04	3.31	−3.06	−27.64	17.37	0.27	−11.538
PC10	−40.06 *	136.24 **	−4	−30.1	0.43	−3.13	96.6 *	−33.93	−2.49	59.417
PC11	43.16 *	−23.7	−1.44	8.41	7.99	0.52	17.06	7.24	8.52	36.917
PC12	36.75 #	6.33	1.95	−1.73	−1.13	2.01	42.27	0.55	0.99	45.827
PC13	0.08	−1.22	−2.67	−3.6	−0.47	−0.27	−3.04	−6	−0.29	−7.337
Conditional R^2^	0.759	0.747	0.632	0.643	0.816	0.514	0.474	0.672	0.816	0.726
Marginal R^2^	0.016	0.010	0.006	0.010	0.004	0.016	0.015	0.008	0.003	0.010

FL: the sum of 3-FL and 2′-FL; SL: the sum of 6′-SL and 3′-SL; LT: the sum of LNnT and LNT; sum: the sum of all the six kinds of HMOs; ‘**’ *p* < 0.01, ‘*’ *p* < 0.05, ‘#’ *p* < 0.1.

**Table 6:** Top five nutrients within PCs significantly correlated with HMO (and factor loadings).

Principal Components	Top1	Top2	Top3	Top4	Top5
PC1	ferrum	kalium	manganese	calcium	magnesium
	0.903	0.866	0.821	0.760	0.700
PC4	plant protein	plant oil	plant calcium	animal calcium	animal fat
	0.943	0.915	0.904	0.877	0.851
PC5	gamma tocopherol	delta tocopherol	beta tocopherol	C18:3 n linoleic acid	C18:3 total linoleic acid
	0.968	0.943	0.768	0.726	0.723
PC7	milk	lactose	selenium	milk calcium	phosphorus
	0.919	0.899	0.435	0.362	0.354
PC10	supplement	vitamin B2	vitamin B1	alpha tocopherol	total choline
	0.883	0.782	0.667	0.548	0.244
PC11	total carotene	vitamin A	vitamin C	lutein and zeaxanthin	vegetable
	0.787	0.781	0.750	0.732	0.704
PC12	cookie	vitamin B3	cuprum	seasoning	potato starch
	−0.538	0.495	−0.418	0.407	0.390

The full table of PCs and nutrients is available in Supplementary Table S3.

## Data Availability

The data presented in this study are available on request from the corresponding author. A data sharing agreement will be requested.

## Supplementary Materials

The following supporting information can be downloaded at: https://www.mdpi.com/article/10.3390/nu14194131/s1, Table S1: diet between secretor and non-secretors; Table S2: diet in different stages; Table S3: PCA and diet.
